# A systematic literature review of the ethics of conducting research in the humanitarian setting

**DOI:** 10.1186/s13031-020-00282-0

**Published:** 2020-05-24

**Authors:** William Bruno, Rohini J. Haar

**Affiliations:** 1 Department of Emergency Medicine, University of Southern California, Keck School of Medicine, Los Angeles, USA; 2 Division of Epidemiology and Biostatistics, School of Public Health, Research Fellow, Human Rights Center, School of Law, University of California at Berkeley, Berkeley, USA

**Keywords:** Humanitarian crisis, Conflict, War, Ethics, Research, Disasters, Aid

## Abstract

**Background:**

Research around humanitarian crises, aid delivery, and the impact of these crises on health and well-being has expanded dramatically. Ethical issues around these topics have recently received more attention. We conducted a systematic literature review to synthesize the lessons learned regarding the ethics of research in humanitarian crises.

**Methods:**

We conducted a systematic review using the Preferred Reporting Items for Systematic Reviews and Meta-analysis (PRISMA) guidelines to identify articles regarding the ethics of research in humanitarian contexts between January 1, 1997 and September 1, 2019. We analyzed the articles to extract key themes and develop an agenda for future research.

**Results:**

We identified 52 articles that matched our inclusion criteria. We categorized the article data into five categories of analysis: 32 were expert statements, 18 were case studies, 11 contained original research, eight were literature reviews and three were book chapters. All included articles were published in English. Using a step-wise qualitative analysis, we identified 10 major themes that encompassed these concepts and points. These major themes were: *ethics review process* (21 articles, [40.38%]); *community engagement* (15 articles [28.85%]); *the dual imperative*, or necessity that research be both academically sound and policy driven, *clinical trials in the humanitarian setting* (13 articles for each, [25.0%)]; *informed consent* (10 articles [19.23%]); *cultural considerations* (6 articles, [11.54%]); *risks to researchers* (5 articles, [9.62%]); *child participation* (4 articles [7.69%]); and finally *mental health*, and *data ownership* (2 articles for each [3.85%]).

**Conclusions:**

Interest in the ethics of studying humanitarian crises has been dramatically increasing in recent years. While key concepts within all research settings such as beneficence, justice and respect for persons are crucially relevant, there are considerations unique to the humanitarian context. The particular vulnerabilities of conflict-affected populations, the contextual challenges of working in humanitarian settings, and the need for ensuring strong community engagement at all levels make this area of research particularly challenging. Humanitarian crises are prevalent throughout the globe, and studying them with the utmost ethical forethought is critical to maintaining sound research principles and ethical standards.

## Background

Defined as both natural and man-made disasters, along with both acute and chronic conflicts, humanitarian crises threaten the lives and livelihoods of over 131 million people in the world today [1]. With more than 68.5 million people currently displaced, 25.4 million of whom are refugees outside their country of origin, the global community is witnessing urgent humanitarian issues that are crossing borders and impacting even those states and communities once thought immune [2, 3]. Humanitarian aid is the impartial, independent and neutral delivery of services to populations in immediate danger [4]. Since the end of World War II, the humanitarian aid sector (in the form of health services, water and sanitation services, nutritional goods and security) has grown tremendously [5].

With expansion in humanitarian aid delivery and the deepening awareness that humanitarian crises can destroy health systems and have long-term impacts on public health, ensuring that the services provided are effective and acceptable is crucial. Following several highly publicized failures of the humanitarian community, veteran humanitarians from across the spectrum of governmental and non-governmental organizations have attempted to improve humanitarian response [6]. Initiatives such as the Sphere Project and others aimed to create minimum standards and evidence-based protocols for the delivery of five core components of humanitarian response—water supply and sanitation, nutrition, food aid, shelter and site planning and health services [7]. Over the past several decades, a key component of the assessment process has been conducting formal monitoring, evaluation and research on humanitarian aid delivery. Studies ranging from randomized control trials to population surveys and qualitative assessments evaluating the full spectrum of humanitarian aid delivery have burgeoned [8].

Parallel to the increase in professionalization of humanitarian aid, the public health community has been grappling with how to ensure that research on vulnerable populations is conducted ethically and with a focus on the rights and best interests of the community. Spurred by a backlash to unchecked human experimentation carried out through the twentieth century during World War II and the decades afterwards, there is more recognition of the critical importance of considering research ethics, particularly when studying vulnerable populations [9].

Few populations are as vulnerable to the potential adverse ethical challenges of research as those experiencing a humanitarian crisis [10]. Faced with weak government protections, disrupted health systems, insecure living conditions, and unreliable food and unsafe water, disaster-affected populations can be particularly at risk of inadequate consent processes and coercion. Furthermore, humanitarian emergencies require timely evaluation and management, making traditional ethics review—typically a protracted process—impractical [11–13]. These unique challenges, along with underdeveloped oversight and regulatory bodies of host countries and international mechanisms, make ethics considerations a crucial but difficult task in humanitarian research [14, 15].

Despite increasing interest and an expanding literature base, there has been limited formal synthesis of the existing published data around the ethical issues of research in the humanitarian setting. We conducted a systematic review to (1) identify ethical issues surrounding research in humanitarian settings, (2) assess how these issues are managed in these unique circumstances and (3) develop an agenda for major issues that will require further discourse.

## Methods

We conducted a systematic review using the Preferred Reporting Items for Systematic Reviews and Meta-analysis (PRISMA) guidelines [16]. The PRISMA checklist has been provided as Supplementary Table 1. Articles relevant to research ethics in the humanitarian setting were identified and analyzed. We chose to limit the search to articles published after January 1, 1997, when the initiation of the Sphere project marked a paradigm shift in how humanitarian aid was envisioned and carried out. This allows for review of nearly 25 years of literature, therefore spanning a wide swath of potential ethical research. We used the Sphere project dates because it included explicit language highlighting the need for evidence-based practices, which would require significant augmentation in research efforts to provide such an evidence base [7]. Our search included articles published as late as September 1, 2019, when this study was first undertaken.

### Search strategy

**Fig. 1:**
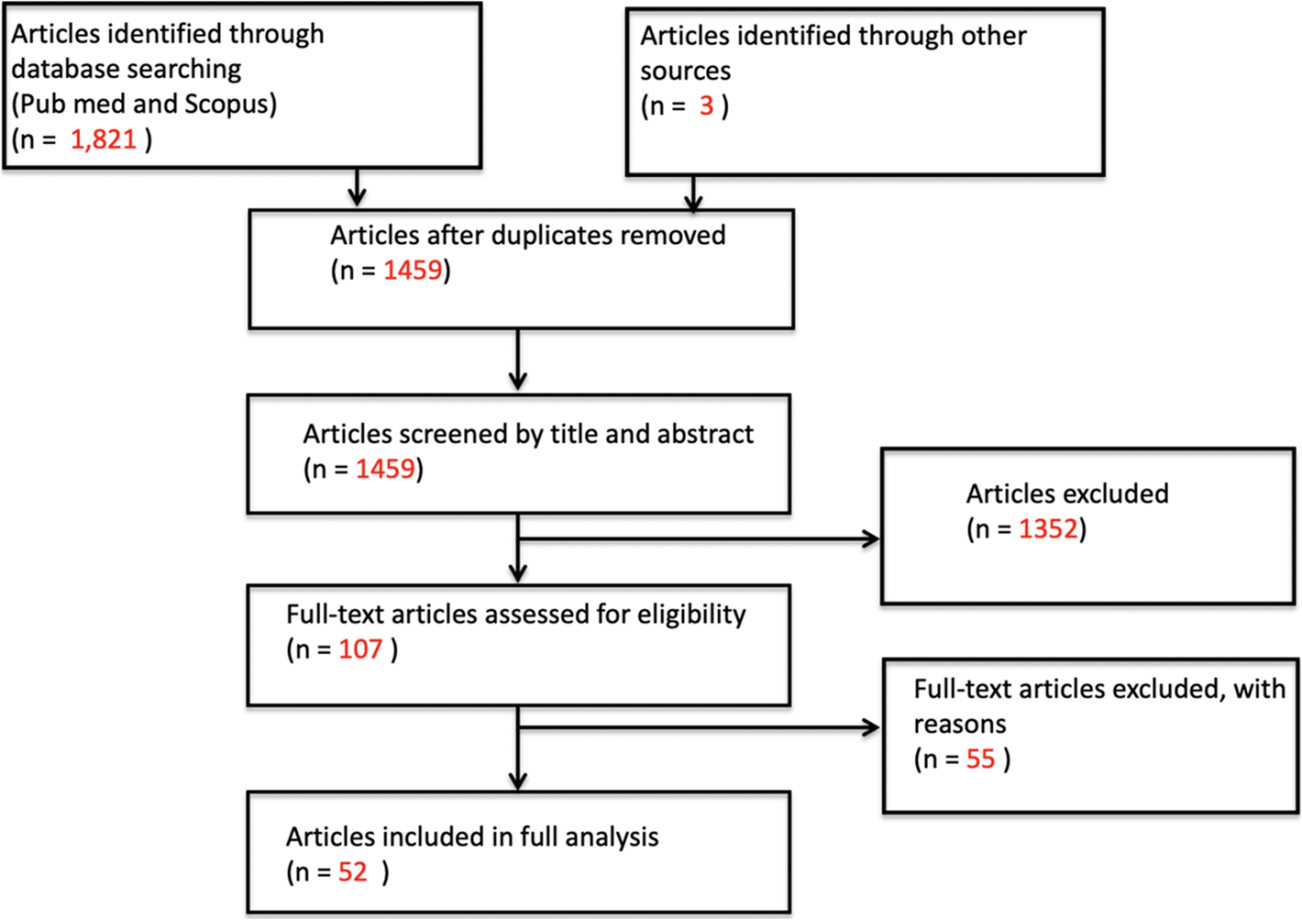
Stages of Systematic Literature Review Utilizing PRISMA Guidelines

We searched PubMed and Scopus for articles with significant discussion of the ethical issues of humanitarian research ethics. After a qualitative assessment of relevant keywords, we identified all pertinent articles based on the following terminology categories (articles could be in any language): (1) humanitarian settings (terms such as *humanitarian, global health, disaster, emergency and/or conflict), (2) ethics (terms such as ethic(s), bioethics, human rights and/or rights) and (3) research type (terms such as research, program evaluation, monitoring and evaluation and/or investigation). The full search strategy and MeSH terms can be found in the**Appendix**.* The initial search results of 1459 articles underwent a title and abstract review followed by a full text review by two different authors (WB and RH) (Fig. 1). A priori inclusion criteria included the 22-year timeframe mentioned above and selected for articles with robust discussion of ethical issues in the context of conducting research in humanitarian settings. Any article deemed by both reviewers to contain only a superficial mention of ethical issues and to not substantively (1) discuss ethics or (2) focus on research (3) in the context of humanitarian settings was excluded from the final analysis. Ethics was defined broadly as engagement with specific research ethics, as well as human rights issues, and other non-formal discussions of right versus wrong and other moral concepts. Research was defined as discussions including any types of data collection including quantitative and qualitative, as well as data collection for monitoring and evaluation for other programmatic and academic purposes. Humanitarian settings included diverse contexts including conflict and post-conflict states, post-natural disaster settings and refugee camps that requires specific interventions to prevent large scale suffering of the populations. Two authors (WB and RH) reviewed the final list of articles meeting the inclusion criteria.

### Analytical methods

We used a modified meta-ethnographic approach to inductively identify key concepts and synthesize the major themes [17]. We chose the meta-ethnographic approach as it has been shown useful in other systematic reviews of qualitative health literature in that it utilizes an inductive approach that can account for differences in methodology and focus, and has the potential to provide a higher level of analysis and generate new research questions [18–20]. We conducted three steps of analysis: (1) Identifying original concepts and ideas from each paper that related to cross-cutting themes; (2) synthesizing these ideas into cross-cutting themes; and (3) identifying major themes. These steps are outlined in Table 2. Original concepts were topics discussed in each paper, which the authors felt had some relevance to this paper’s focus on humanitarian research ethics. Cross-cutting themes were key concepts that were identified in at least two different articles. We assessed how the cross-cutting themes may fall into broader overarching ideas and coded these into related non-mutually exclusive groups we termed major themes. The synthesis process of extracting these major themes was one of reciprocal translation and constant comparison of concepts across studies. The process elucidated tensions and areas for future research within each major theme, as shown in Table 2. Any disagreements on the analysis were resolved with discussion and consensus.

This research, based on previously published literature, did not meet criteria for Institutional Review Board approval.

## Results

**Fig. 2:**
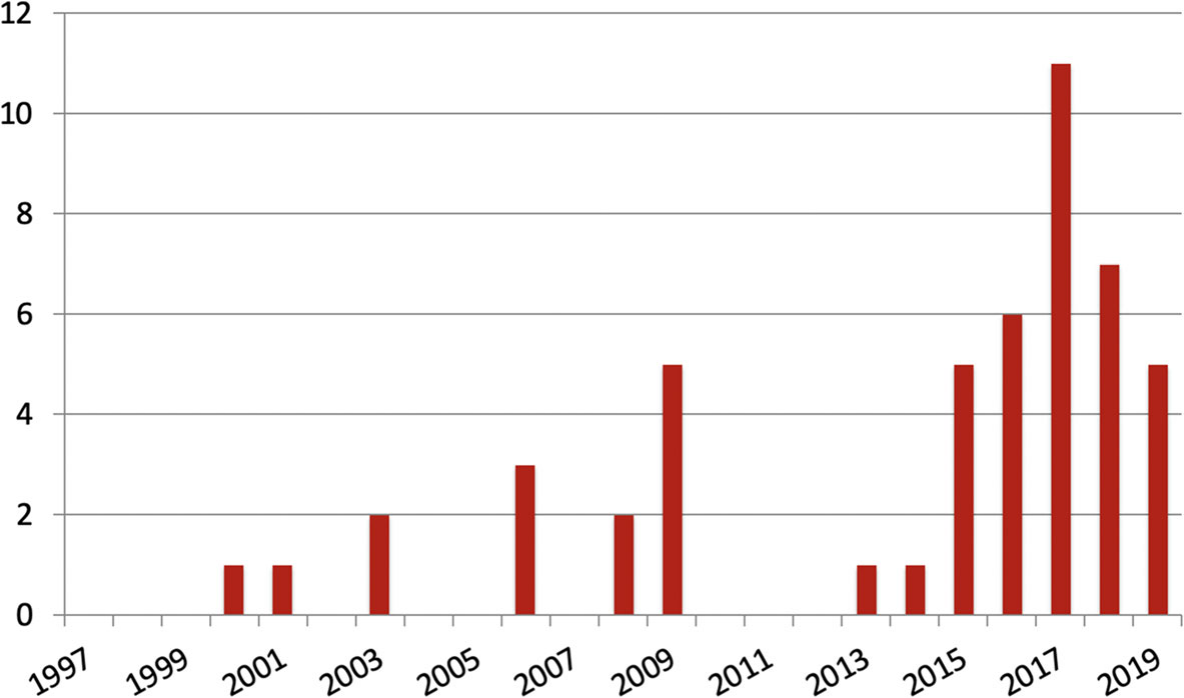
Included articles by publication date

Of the 1459 unique articles resulting from our search terms, 52 matched our inclusion criteria (Table 1: List of Included Articles). The articles took the shape of five non-mutually exclusive categories of analysis: 32 were expert statements, 18 were case studies, 11 contained original research, eight were literature reviews and three were book chapters. All included articles were published in English. Thirty-four of the 52 (65.38%) articles were published in 2015 or later, ten between 2007 and 2014, and eight were published in the 1997–2006 decade (Fig. 2). Of the 52 articles included for final analysis, 23 were published by international teams (meaning that they were comprised of members from at least two different countries), 12 were from the United States, six from the United Kingdom, three from Canada, two each form Ireland, Trinidad and Tobago, and Switzerland, and one each from Australia and India.

**Table 1:** List of Included Articles

Title	Author	Year	Ref.	Country
Public health and humanitarian interventions: Developing the Evidence Base	Banatvala et al.	2000	[41]	United Kingdom
Ethics of research in refugee populations	Leaning et al	2001	[71]	United States
Ethical Codes in Humanitarian Emergencies: From Practice to Research?	Black, R et al.	2003	[57]	United Kingdom
The Dual Imperative in Refugee Research: Some Methodological and Ethical Considerations in Social Science Research on Forced Migration	Jacobsen et al.	2003	[59]	International
Are adaptive randomized trials or non-randomized studies the best way to address the Ebola outbreak in west Africa?	Lanini et al.	2003	[51]	International
Is it ethical to study what ought not to happen?	Rennie	2006	[55]	United States
Do aid agencies have an ethical duty to comply with researchers? A response to Rennie	Zachariah et al.	2006	[63]	International
The Ethical Challenges of Field Research in Conflict Zones	Wood	2006	[62]	United States
Fieldwork and social science research ethics	Contractor et al.	2008	[58]	India
Ethical Challenges in Conducting Research in Humanitarian Crisis Situations	Mfutso-Bengo et al.	2008	[50]	International
The control of foreigners as researchers in Thailand	Ditton et al.	2009	[47]	Australia
Real-time Responsiveness for Ethics Oversight During Disaster Research	Eckenwiler, et al.	2009	[24]	International
Ethics of Conducting Research in Conflict Settings	Ford et al.	2009	[48]	International
Ethical considerations of research in disaster-stricken populations	Jesus et al.	2009	[64]	United States
Health Research in Complex Emergencies: A Humanitarian Imperative	Pringle et al.	2009	[60]	Canada
Conducting research in the aftermath of disasters: ethical considerations	O’Mathúna	2010	[23]	Ireland
Reflections on Ethical and Practical Challenges of Conducting Research with Children in War Zones: Toward a Grounded Approach	Wessells	2013	[61]	United States
Conducting surveys in areas of armed conflict	Mneimneh et al.	2014	[68]	Unites States
Use of a bibliometric literature review to assess medical research capacity in post-conflict and developing countries: Somaliland 1991–2013	Boyce et al.	2015	[45]	International
Ethics, emergencies and Ebola clinical trials: the role of governments and communities in offshored research	Folayan et al.	2015	[30]	International
Research ethics in the context of humanitarian emergencies	O’Mathúna	2015	[27]	Ireland
Innovations in Research Ethics Governance in Humanitarian Settings	Schopper et al.	2015	[31]	International
“Losing the tombola”: a case study describing the use of community consultation in designing the study protocol for a randomised controlled trial of a mental health intervention in two conflict-affected regions	Shanks et al.	2015	[42]	International
Ethics in Community-Based Research with Vulnerable Children: Perspectives from Rwanda	Betancourt et al.	2016	[43]	International
The Ebola clinical trials: a precedent for research ethics in disasters	Calain	2016	[53]	Switzerland
Managing Ethical Challenges to Mental Health Research in Post-Conflict Settings	Chiumento et al.	2016	[21]	United Kindom
Research as intervention? Exploring the health and well-being of children and youth facing global adversity through participatory visual methods	D’Amico et al.	2016	[65]	Canada
The Challenge of Timely, Responsive and Rigorous Ethics Review of Disaster Research: Views of Research Ethics Committee Members	Hunt et al.	2016	[11]	International
Emergency response in a global health crisis: epidemiology, ethics, and Ebola application	Salerno et al.	2016	[52]	International
Ethics review of studies during public health emergencies - the experience of the WHO ethics review committee during the Ebola virus disease epidemic	Alirol	2017	[38]	Switzerland
Ethical considerations for children’s participation in data collection activitie during humanitarian emergencies: A Delphi Review	Bennouna et al.	2017	[67]	United States
Reflections on the ethics of participatory visual methods to engage communities in global health research.	Black, GF et al.	2017	[44]	International
Challenges in preparing and implementing a clinical trial at field level in an Ebola emergency: A case study in Guinea, West Africa	Carazo et al.	2017	[46]	International
Ethical standards for mental health and psychosocial support research in emergencies: review of literature and current debates	Chiumento et al.	2017	[22]	United States
Research in disaster settings: a systematic qualitative review of ethical guidelines.	Mezinska et al.	2017	[35]	International
Conducting Science in Disasters: Recommendations from the NIEHS Working Group for Special IRB Considerations in the Review of Disaster Related Research.	Packenham et al.	2017	[26]	United States
A Systematic Review of Ebola Treatment Trials to Assess the Extent to Which They Adhere to Ethical Guidelines	Richardson	2017	[36]	United Kingdom
Research Ethics Governance in Times of Ebola	Schopper et al.	2017	[29]	International
Familiar ethical issues amplified: how members of research ethics committees describe ethical distinctions between disaster and non-disaster research	Tansey et al	2017	[33]	Canada
Research ethics and evidence for humanitarian health	O’Mathúna et al	2017	[28]	International
Research in epidemic and emergency situations: A model for collaboration and expediting ethics review in two Caribbean countries	Aarons	2018	[39]	Trinidad and Tobago
Addressing the challenge for expedient ethical review of research in disasters and disease outbreaks	Aarons et al.	2018	[66]	Trinidad and Tobago
Ethical Challenges Among Humanitarian Organisations: Insights from the Response to the Syrian Conflict	Funk et al.	2018	[49]	United States
Research Ethics Committees (RECs) and epidemic response in low and middle income countries	Bain et al.	2018	[40]	International
Ethical Issues in Conducting Research With Children and Families Affected by Disasters	Ferreira et al.	2018	[70]	International
Social value, clinical equipoise, and research in a public health emergency	London et al.	2018	[56]	United States
Individual and public interests in clinical research during epidemics: a reply to Calain: In response to: Calain P. The Ebola clinical trials: a precedent for research ethics in disasters	Rid	2018	[54]	United Kingdom
Health-emergency disaster risk management and research ethics	Chan et al.	2019	[34]	International
Ethical Challenges in Humanitarian Health in Situations of Extreme Violence	Collaborative	2019	[69]	United States
The ethical contours of research in crisis settings: five practical considerations for academic institutional review boards and researchers	Falb et al	2019	[25]	United States
Mention of ethical review and informed consent in the reports of research undertaken during the armed conflict in Darfur (2004–2012): a systematic review	Hussein et al.	2019	[37]	International
Ethics preparedness: facilitating ethics review during outbreaks- recommendations from an expert panel	Saxena et al	2019	[32]	International

### Thematic analysis

The step-wise analysis is presented in Table 2. First order analysis of the articles meeting our final inclusion criteria revealed ideas and issues within the context of ethics related research in humanitarian settings. In the second phase of the analysis, qualitative review of the reports identified cross-cutting themes between the papers, and 10 major themes that encompassed these concepts and points. These major themes in descending order of prevalence were *ethics review process* (21 articles, [40.38%]); *community engagement* (15 articles [28.85%]); *the dual imperative*, or necessity that research be both academically sound and policy driven and *clinical trials in the humanitarian setting* (13 articles for each, [25.0%]); *informed consent* (10 articles [19.23%]); *cultural considerations* (6 articles, [11.54%]); *risks to researchers* (5 articles, [9.62%]); *child participation* (4 articles [7.69%]), and finally *mental health*, and *data ownership* (2 articles for each [3.85%]).

**Table 2:** Summary of Modified Meta-ethnographic Analysis

Original Concepts and Ideas Taken From Articles	Cross-cutting Themes	Tensions and Future Research	Major Theme
Lacking or dysfunctional review boards in LMICs [21] Inability of traditional ethics review during an emergency [22, 23] Real time responsiveness (RTR) ethical review [24] Ethical review challenges in humanitarian setting [25] Challenge of timely, efficient and comprehensive ethical review [11, 26] Traditional ethics review is not suitable to practical realties [27, 28] MSFs ERB during Ebola outbreak [29, 30] Ethics review board specific for MSF [31] Recommendations for improving ethical review [32] Unique set of ethical priorities governing post-disaster research [33, 34] Ethical guidelines revealed two core themes: vulnerability and review process [35] Deviation from normal ethical oversight in disaster setting may be acceptable [36] Historical lack of oversight in humanitarian contexts [37] Uniqueness of ethical review during Ebola outbreak [38] Regional collaboration for ethical review [39, 40]	In-country, local review [21, 30] Timely ethics review during and emergency [11, 22, 23, 29, 31, 35, 38] Question as to whether or not ethics review in the humanitarian setting should have different standards compared with traditional review [27, 28, 31, 33, 35, 36, 38] Collaboration across institutions for ethical review [37, 39]	**Tensions:** Inherent vulnerability makes ethical review processes extremely important. However, the timely nature of humanitarian situations makes traditional ethics review impractical. **Future research/initiatives:** Outline the specifics of ethical review of research in the humanitarian setting, as this process should be unique from traditional ethics review.	*Ethical Review*
Ensuring that communities enjoy maximum benefit of research [41] Case studies on ethical research [42] Engagement with local governments/health authorities [43] “Uncomfortable power dynamic” between researchers and communities [44] Low research output from researchers from LMIC [45] Community engagement to facilitate a clinical trial [46] Ethical entry” and compliance with local cultural norms [21] Utilizing gatekeepers may augment hierarchies of power [22] Thai Government as a gatekeeper via a permit system [47] Community involvement as benchmark for ethical research [48, 49] Risk of cooperating with nefarious authorities [49] Ambivalence about working with authority figures [50] Local stakeholders involvement in ethicial review [32] Ethics committee members view of community engagement [33] Community engagement to avoid “clinical trial exploitation” [30]	Will local populations benefit from the research? [21, 30, 41, 42, 46, 48] Community engagement enhances cultural understanding, which can help reduce harm amongst participants [22, 43, 44, 46, 48] Engagement with local authorities may be necessary, though it has potential unintended consequences on local power dynamics and perceived legitimacy of the researchers [47, 50] Limited capacity of locals to conduct their own research [21, 33, 45, 47, 49]	**Tensions:** There is a need to work with local authorities for both practical and ethical reasons, though there is concern that this cooperation can be seen as legitimizing this authority. This may be undesirable. Without local participation it is unlikely that they will reap the full benefits of the research product. However, including locals in research is inherently difficult. **Future research/initiatives:** Formal post hoc evaluations to help determine if the community did, in fact, benefit from the research product. Strategies to prepare locals for a participatory role in research are needed.	*Community Engagement*
Argument for single arm design over RCT for Ebola clinical trials [46] Clinical equipoise as justification for Ebola Virus Disease RTCs [51] Clinical equipoise justifies research in the humanitarian setting [27] Proposal for adaptive (Bayesian, cluster or step wedge) research [52] An a priori exclusion of pregnant subjects would deprive them of potential benefits of the research [29] Community engagement to avoid “clinical trial exploitation” [30] The individual vs. the collective interest complicates clinical trial ethics [53] Lack of focus on positive obligations of researchers toward participants [54] Systematic review demonstrates deviation from normal ethical oversight during clinical trials [36] Study design has ethical implications [38, 55] Refutation of a priori right to unvalidated clinical interventions [56]	Clinical trials where there is no known treatment for a catastrophic disease [27, 29, 30, 46, 51, 52] Oversight might be necessary to avoid exploitation [30, 36] Clinical equipoise and justification of RCTs [38, 54, 56]	**Tensions:** It is unclear which type of clinical trial is most appropriate in the humanitarian setting. **Future research/initiatives:** Review or meta-analysis to evaluate the best methodology for clinical trials in the humanitarian setting.	*Clinical Trials*
Collaboration between academics and practitioners [41] Conflict in between researcher’s objectivity, and humanitarian’s advocacy [57] Researchers simultaneously participating in relief efforts [58] Research should be both academically sound and action driven [11, 59] Research is justified insofar as it is not compromising relief efforts [60] MSF’s refusal to participate in research on treatment rationing [55] The evolution form pure researcher to researcher-practitioner [61] Justification for research in conflict setting [62] Explanation of MSFs ethics of studying HIV medication rationing [63] Generally limited resources in these settings [64] Effective research design might inhibit optimal treatment initiatives [53]	Research at the expense of intervention, as these two goals may come in conflict [53, 57, 58, 60, 62, 64] Collaboration between NGOs and academic institutions [41, 59, 61] Discussions on the ethics of researching policies, which may be in conflict with humanitarian principles [55, 63]	**Tensions:** Research should be both academically rigorous and practical. Given that humanitarian response is grounded in specific principles is it ethical to research policies that fall outside of these principles? **Future research/initiatives:** Strengthen the relationships between humanitarian aid groups and academic institutions.	*Dual Imperative*
Reassessing a participant’s consent during the experiment [44] Informed consent in the setting of a particularly fatal disease [46] Use of a “gatekeeper” when seeking informed consent [21] A more nuanced view of consent might be more suitable for emergencies [21] More flexible approach to consent [22] “Humanitarian misconception” [27] Challenges of consenting children [65] Consent during disasters may be coercive [23] Exclusion of groups may undermined justice [38] Regional collaboration for ethical review [66]	Dynamic consent [38, 44] Participants may find it difficult to separate consent for research from receiving aid [27, 38, 46] Use of gatekeepers for attaining consent [21] Acceptance of less rigid consent procedures in the humanitarian setting [21, 22] Forcing participants to relive trauma for the purpose of research raises ethical questions [44, 65] Unavoidable coercion [23]	**Tensions:** May be necessary, for both practical and cultural reasons, to obtain consent for participation through an intermediary, which is in conflict with principle of autonomy. **Future research/initiatives:** Outline the specifics of consent for participation in research in the humanitarian setting.	*Informed consent*
Child participation in conflict with local norms [67] Use of gatekeepers for informed consent [21] Research teams with local knowledge [50, 57] Thai permit system vs. western funding regulations [47] Research teams with local knowledge [50]	Cultural relativism [67] Gatekeepers and cultural liaisons [21, 47] Cultural competency and humility [50, 57]	**Tensions:** Much of the literature puts a premium on respecting cultural norms. There is also an understanding that these norms may be in conflict with accepted ethical principles. **Future research/initiatives:** Identify core principles or universal research ethics, which supersede cultural norms in so far as these norms come in conflict with the former, and thus justify their disregard.	*Cultural Considerations*
Discussing sensitive topics can put researchers at risk [21] Unethical to put a researcher in a dangerous position without clear adequate forethought [68, 69] Researchers may witness horrific events, and should consent to these risks [23, 49]	Need for formal protocols for responding to threatening situations [21, 68] Concent of researcher [23, 49]	**Tensions:** Working in humanitarian contexts comes with risk, and minimizing this risk is an ethical imperative. **Future research/initiatives:** Formal protocols for minimizing the risk to researchers.	*Risks to Researchers*
Consensus that children should be involved as research participants [67] Researchers must anticipate urgent issues [43, 70] Consenting children to relive trauma for the purpose of research [65]	Fundamental right that children be allowed to participate in research [43, 67, 70] Challenges of consenting children [65, 70]	**Tensions:** Children are particularly vulnerable but systematically excluding them from research participation could be unethical. **Future research/initiatives:** Clear guidelines for determining when the risks of including children outweigh the benefits.	*Child participants*
Concern around the extraction of knowledge from disaster stricken areas [57] Data sharing as ethical imperative [45]	Data ownership as it relates to who benefits from research [45, 57]	**Tensions:** Potential extractive relationship in which the data produced in low-income countries is circulated only in high-income countries. **Future research/initiatives:** Standardized policies for data ownership	*Data Ownership*
Stigmatization makes conducting research on mental health in LMICs particularly difficult [21] Friction between procedural ethics and ethics in practice vis-à-vis mental health [22]	Local stigma towards mental health complicates research on these topics [21, 22]	**Tensions:** Given pervasive stigma, studying mental health might put the subjects as well as researchers at risk. **Future research/initiatives:** Develop strategies to dispel stigma and misconceptions about mental health.	*Mental Health*

### Ethical review

Discussion of the ethical review process was the most commonly identified theme, with 21 articles having a substantive focus on this [11, 21–40]. Independent ethics review prior to the start of a study is a core component of research ethics. Tansey et al. conducted a survey of ethics review board members with experience in reviewing research ethics in disaster settings. Their results suggest a general feeling that research in this setting is not only of particularly high social value, making it a desirable pursuit, but also necessitates a higher level of justification due to the inherent vulnerability of the research subjects [33]. There is also general agreement that the innate fluidity and urgency of humanitarian situations make swift and efficient ethics review of paramount importance [11, 25, 29]. Hunt et al. report, “where research is launched in response to a sudden-onset disaster such as an earthquake or hurricane, researchers may need to initiate their protocols quickly in order to answer research questions pertinent to the acute phase of the disaster response” [11]. However, as mentioned above, the particular vulnerability of the subjects being studied leads many research ethics committees to automatically identify humanitarian research as requiring “the highest level of stringency”. On the other hand, framing research as “needs assessments” and/or “monitoring and evaluation,” which is often done in evaluating aid needs and programs, may act to sideline rigorous ethical review and jeopardize the well-being of the recipient population [11]. This contradiction of values makes ethical review of humanitarian research particularly challenging.

Authors suggested strategies to mitigate the inherent challenges of ethics review in this setting [25]. For example, Hunt et al. suggest pre-approved research protocol templates which can be quickly customized for use in individual emergencies [11]. Eckenwiler et al. propose what they refer to as ‘real-time responsiveness,’ which is an iterative strategy of constant dialogue between ethics reviewers and researchers while studies are being conducted [24]. Given the potential for misstep in an expedited initial ethics review, Chiumento et al. describe the utility of a post-research ethical audit. The authors explain how this could help to evaluate “procedural ethics against in-practice realities”, which could help inform future studies [21]. Ethical analysis after data collection may also offer the added benefit of offering lessons on the review and practice process to the reviewers and researchers.

Our results highlighted the particular case of how the humanitarian aid agency Médecins Sans Frontières’ (MSF), who conducts substantial research in humanitarian settings, has devised an independent Ethics Review Board (ERB). The ERB utilizes several of the strategies mentioned above such as pre-approved protocols, engaging in ongoing dialogue between researchers and the ERB and conducting post-research evaluations [29, 31]. Saxena et al. reported on a joint panel conducted by the WHO and the African Coalition for Epidemic Research, Response and Training. The authors outline the group’s recommendations for “rapid and sound ethics review”, which includes “preparing national ethics committees for outbreak response; pre-crisis review of potential protocols; multi-country review; coordination between national ethics committees and other key stakeholders; data and benefit sharing; and export of samples to third countries” [32]. Indeed, as Mezinska et al. point out in their systematic review of ethical guidelines, most of the analyzed documents included in their report did “not attempt to give researchers and other stakeholders a comprehensive overview of how to proceed ethically in all types of research and in all types of disasters”, which the authors see as problematic given that “disaster research is unavoidably context and time sensitive, making generalized guidance less applicable” [35].

### Community engagement

Substantive involvement of the community being studied was identified as an imperative for researchers and a major theme of discussion in 15 articles [21, 22, 30, 32, 33, 41–50]. It was generally agreed that active participation is necessary in order to fulfill the ethical requisite that research be of use to the community being studied (also known as beneficence) [22, 48, 50]. As Chiumento et al. identified in their systematic review of mental health literature, the right to participate in research can be viewed as a basic right in and of itself, insofar as it relates to other rights such as self-determination and autonomy [22]. One important strategy described was involving local community health and government officials in an effort to maximize community support [43]. More practically speaking, this effort can help limit potential for a community’s misunderstanding of research, which can jeopardize a project’s legitimacy and undermine its acceptance [46]. Early involvement of community actors, potentially via consultation during study protocol design or community meetings, was suggested [21, 42].

The discussions within the articles suggest that community involvement also involves strengthening local institutions, effectively improving their ability to conduct their own research [21, 22]. Despite being recognized as an important component of ethical research, it was generally agreed that there is a critical shortage of local capacity to carry out studies, particularly in post-conflict zones where formal institutions are often eroded [45, 47]. In their study on the research capacity of Somaliland, Boyce et al. identified potential harms of a “dominance of authors from [High-Income Countries]” [45]. They explain that, for example, the unrelatability between researcher and subject could lead to a reduced relevance of the research question.

Despite the agreement for “a set of practices that help researchers establish and maintain relationships with the stakeholders to a research program”, Tansey et al. discuss some of the inherent challenges in community participation. Particularly when conducting disaster research, the practicality of including locals can be difficult when “you don’t know when the disaster is going to hit. .. so it would be hard to set up community approvals and engagement beforehand” [33]. Furthermore, lack of adequately trained researchers and poor local infrastructure are perennial problems [45]. While ethically desirable, partnering with the local community may, in many circumstances, often prove practically prohibitive.

While including local authorities in research may seem prudent on face value, as discussed in the section on cultural considerations, these articles make clear the potential for ethical ambiguity when dealing with such actors [47, 49]. For example, in a civil war context, researchers may hope to adhere to humanitarian principles of impartiality to ensure access to participants and safety for researchers [49]. Furthermore, as Funk et al. describe in their evaluation of the response to the Syrian conflict, remaining impartial can be impossible. One respondent explained, “You have to understand that even though we declare ourselves as a non-biased health organization with no political standing, the mere fact that we are not ‘pro-government’ makes us [perceived as] ‘the enemy’ and ‘anti-government’” [49].

### The dual imperative

Thirteen articles discuss what humanitarian researchers refer to as the ‘dual imperative,’ which is the inherent tension between ensuring that research is both academically sound and practically relevant [28, 41, 53, 55, 57–64, 71]. Despite the inherent challenges in humanitarian research, the general consensus is that it is justifiable insofar as it is needs-driven and not at the expense of humanitarian action [60]. However, as researchers attempt to construct sophisticated research and attract funding, there is a move toward a greater level of academic sophistication [59]. On the individual level, a member of a humanitarian response team may feel responsibilities as both service provider and researcher [58, 61]. Wood, in her description of experiences researching conflict zones in El Salvador, describes an inevitable self-inquiry of why this research is worth pursing at the expense of a purely humanitarian medical relief mission. She concludes that her role as a researcher was justified in that a sound understanding of conflict is necessary for its abolishment. Wood does, however, concede that this conclusion may be predicated on the nature of the “relatively benign and coherent conditions” of her work. Specifically, she “did not have to make a decision whether or not to intervene to attempt to prevent or mitigate an attack on civilians.” She “did not have to decide how to leave an area under attack at short notice, retreating with one force or seeking shelter from another.” She was “never faced with direct threats [insisting] that [she] turn over material [she] had gathered” and did not have “to judge how far to press respondents about violence they had suffered or observed because of the focus of [her] research.” The implication was that had she been faced with one of these more charged situations, her resolve in the justification of research would be challenged. In fact, she ends her discussion by stating that “conditions in many civil wars simply preclude ethical field research” [62].

Another related point of contention identified in our search is a disagreement that arose between a researcher and aid agency. Due to an overtaxed and under resourced system, the Democratic Republic of Congo had engaged in rationing of AIDS medications. Rennie, a global health researcher, had intended to study the community attitudes toward this practice [55]. Feeling rationing medications to be unethical, the aid agency Médecins Sans Frontières (MSF), specifically MSF-Belgium, wrote a letter informing Rennie that they would not support his investigation [55, 63]. They expressed concern that the research might be a form of acquiescence to the practice of drug rationing, which they see as antithetical to the humanitarian mission [63]. This tension between assessing an existing program and unintentionally bringing legitimacy to it is one of many practical conflicts in humanitarian research that requires further consideration.

### Clinical trials in the humanitarian setting

Given that clinical trials are considered imperative for investigating medical interventions, many researchers advocate for these types of studies in the humanitarian setting. Thirteen articles explore the ethics of conducting clinical trials in the humanitarian setting [27, 29, 30, 36, 38, 46, 51–56, 63]. Lanini et al. make the point that the principle of clinical equipoise should apply in the humanitarian setting as in any other, making randomized controlled trials (RCTs) the most ethical way to conduct research in this situation, using the recent Ebola outbreak and subsequent drug trials to illustrate their point [51]. With respect to Ebola, Perez et al. make the claim that, given the lethality of the disease, not including pregnant women and children (two groups often excluded from trials on grounds of inherent vulnerability) in Ebola trials is unethical [46]. This, however, presupposes a benefit to the experimental arm of a hypothetical trial, which would violate the principle of clinical equipoise and thus Lanini et al.’s justification of clinical trials outlined above [51]. Salerno et al. argue that the unique circumstances of conducting research in humanitarian settings necessitates that the researcher be less stringent in terms of study design. As the authors explain, “the recipients of experimental interventions, locations of studies, and study design should be based on the aim to learn as much as we can as fast as we can without compromising patient care or health worker safety, with active participation of local scientists, and proper consultation with communities” [52].

Again, with a focus on the recent Ebola outbreak, Calain makes an argument that insistence on RCTs, in which, by definition, one group of participants will be denied the experimental treatment, equates to a preference toward a collective interest (i.e. societal) over the individual (i.e. the patient) which could violate the basic principle of beneficence [53]. For Calain, in the face of a catastrophic illness like Ebola, randomization of interventions is seen as a “tragic choice” for humanitarian workers [53]. Furthermore, as Schopper et al. explained, there is justifiable concern that clinical trials during such an epidemic, which require significant amounts of resources and planning, would detract from the crucial work of directly caring for patients in a resource limited setting [29].

### Informed consent

Like formal ethical review, informed consent is another core component of modern research ethics and was separately discussed in ten articles [21–23, 27, 37, 38, 44, 46, 65, 66]. Our results highlight several unique considerations when contemplating informed consent in humanitarian settings. For example, Western norms of written consent might be impossible if research is carried out in a population with low literacy rates or when written consent can violate the need for complete anonymity or expeditious research [21, 22, 44]. Controversy surrounding traditional ideas of informed consent were highlighted by Chiumento et al. in their literature review [22]. The authors explain that despite the general consensus that informed consent was central to ethical research, there were some authors who emphasized a more informal process that considered “consent as a partnership between researchers and participants” [22]. Some authors surveyed in the study supported flexibility in informed consent by utilizing a “consent framework” that presumably ensures norms such as autonomy and capacity, but allows some latitude for the researcher to adapt to the circumstances. Germane to this point is what Black et al. describe as “dynamic consent”—where a participant’s willingness to be involved in a project is constantly reassessed [44].

Chiumento et al. explain that because of cultural norms, the typical processes of consent may be undesirable or even impossible [21]. In their case study of research conducted in a post-conflict setting in South Asia, they explain that the procurement of informed consent first required permission from gatekeepers (i.e. household males and village elders) [21]. They outline the concept of negotiated consent in which collaboration with researchers helps to distil what exactly culturally specific consent would look like and proceed with an ad-hoc consent process [21].

Our results suggest that special attention be paid to informed consent during clinical trials conducted in the humanitarian setting [29, 46, 51]. Particularly illustrative is the idea of informed consent for experimental therapies during the Ebola outbreak in West Africa in 2014–2015 [46]. Authors raise the question as to whether or not informed consent, free of coercion, can really be possible when potential subjects are faced with such a deadly disease [23].

The use of participatory visual methods (PVM) poses specific challenges with regard to informed consent. The methods ask researchers to encourage subjects to engage in creative forms of communication and expression, such as drama, photography, film, drawing, design, creative writing and music. The products can then be used to engage the community and answer research questions.

However, as participants are synthesizing novel content during the study, and are often encouraged to draw on traumatic experiences as inspiration for this content, fully informed consent is impossible. This is because neither participants nor investigators can completely anticipate which direction their facilitated creative endeavors might turn [44, 65]. This type of research may require more creative or dynamic forms of consent such as frequent check-ins with participants, or “dynamic consent”, as described above.

### Cultural considerations

The importance of strong appreciation, humility, and understanding of local culture was discussed to a robust degree in six articles [21, 47, 50, 57, 64, 67]. As Black et al. explain, research can only be legitimate if it accepts the people as central actors [57]. They describe how community and cultural dynamics may be vital to ensuring that the products of research not be utilized in perverse ways [57]. The authors explain that analyzed and interpreted data on a particular population could be of strategic value to belligerents in a conflict setting [57]. This notion presents an obvious ethical challenge as it has the potential to make researchers active participants in conflict or surveillance. One may conclude that the solution is for researchers to refuse to share data with any local authorities. This, however, conflicts with what Ditton et al. refer to as a vital aspect of ethical field research, namely “the importance that the researcher has an appropriate relationship with the legitimate gatekeepers [and policy makers] of a field site” [47]. As the authors note, local authorities may have perfectly legitimate reasons for demanding cooperation and transparency from researchers. For example, in Thailand, government control of researchers might be justifiable since they espouse it as necessary to ensure that the local population is the ultimate beneficiaries of the research produced within their communities. The government, being responsible for the public’s well-being, argues that having some control over research activities is necessary for them to meet this responsibility [47].

Despite general agreement about the importance of respect for local customs, there is more ambivalence toward which, if any, customs might justifiably be ignored. Bennouna et al. in their survey of researchers explain that 15% of respondents did not believe that local attitudes should be taken into account when deciding on including children in a study, because “what if they tell us not to listen to children?” implying that local norms should not preclude children from having a right to be heard [67]. In contrast, Chiumento et al. suggest “that ethical conduct of research does not equate to importing cultural norms.” The authors continue to describe a common “ethically charged dilemma” in which consent or access to participants first requires permission from a “gatekeeper.” Cultural norms may dictate that (often male) household or community leaders are to make decisions in terms of participation and access to research, depriving some members of the community of basic “ethic and human rights norms” such as autonomy and the right to participate or refuse [21]. These points highlight an unanswered question regarding the universality of ethical principles.

Not only might respect for cultural norms be inherently ethically desirable, but it may also be important for ensuring community participation. As Mfutso-Bengo et al. explain, respect for cultural norms may be necessary “to ensure active community involvement as the community does not perceive overt threats to their way of life” [50]. Balancing fundamental ethical principles of inclusion and autonomy with cultural norms, the articles agree, requires deep cultural understanding.

### Risks to researchers

Five of our included articles discuss the potential risk to researchers working in a humanitarian setting [21, 23, 49, 68, 69]. With the inherent instability of many of these contexts, Chiumento et al. summarize the wide range of potential risks to the wellbeing of researchers, stating that “threats to physical safety; risk of psychological distress; potential for accusations of improper behavior; and increased exposure to everyday risks such as infectious illnesses or accidents” must be recognized [21]. The very nature of conducting research in disaster settings exposes researchers to the potential of witnessing “human carnage and physical destructiveness” [23]. While researchers have personal decision-making responsibilities, host organizations must also acknowledge their obligations to provide security and mitigate risks while ensuring the researchers are fully informed of potential dangers [23, 69].

### Child participation

Child participation in research was discussed in four articles [43, 65, 67, 70]. There was a general consensus that despite being particularly vulnerable, researchers had an ethical responsibility to include children in their studies. This action is necessary, the authors conclude, in order to ensure that children’s voices are heard and that they are not excluded from potential benefits of the research [67].

D’Amico et al. explain “researchers need to develop specific approaches that ensure children understand the benefit of participating voluntarily in research and that consent is informed and an ongoing process” [65]. The challenge, however, as the authors explain, is that through research, particularly qualitative forms such as PVM, “dangerous emotional terrain” might be breeched [65]. The implication is that it is difficult to know whether anyone can fully consent to these unforeseen emotional responses, especially children.

### Data ownership

Two articles describe the unique ethical concerns surrounding data ownership when conducting research in the humanitarian setting [45, 57]. Often, none of the researchers in question are from the communities being studied, so the potential ethical pitfalls of an abusive extractive nature of data collecting might be created [45]. The concern arises when researchers from high-income countries collect data on lower income communities and the ultimate benefits are seen in the former [57].

### Mental health

Mental health research, which was discussed in two articles, has some unique features, which create special ethical issues [21, 22]. For example, Chiumento et al. describe how community mistrust, stigma and paranoia can be particularly significant with regard to mental health, complicating mental health research [21]. There is also a particular importance for confidentiality and anonymity during mental health research given the potential for discrimination and stigmatizing behavior [22].

## Discussion

With the drive toward professionalization of humanitarian practice comes a need to develop a strong evidence base. While the latter half of the twentieth century has seen promising trends in favor of ethical standards for research, the unique conditions of humanitarian work and the particular vulnerabilities of the communities being studied makes exploration of humanitarian research ethics imperative. The time-sensitive nature of the work in combination with complex cultural and security dynamics makes conducting research in the humanitarian setting inherently difficult from an ethical perspective.

Efforts to better understand the nexus between research and humanitarian emergencies are expanding. Other research, including an ongoing review of ethics of humanitarian research and more focused analyses of ethics among specific crises will service to expand this knowledge base [72]. We hope that this paper, representing a broad review and meta-ethnographic analysis of ethical issues in research over more than two decades, strengthens ethical processes and decision making in the humanitarian sector.

Among the 52 articles included in the analysis, 10 major themes regarding the ethics of humanitarian research were extracted for future analysis. In our qualitative analysis of the articles, we found a general acceptance by authors that the increased vulnerabilities of crisis-affected populations lead to several unique issues. Though identified and described in our search, many of these issues have yet to be adequately resolved in a way that might be useful to further researchers. For example, with regard to respect for local cultural norms, our results highlight a unique conflict between a cultural or political demand to share research with a local authoritative body and moral or ethical apprehensions to do so [47, 57]. Authors identified both acceptable and unacceptable reasons for an authoritative body to demand access to research [47, 57]. The researcher must then decide whether they cooperate with authorities by sharing products of their research, and risk being complicit in less socially desirable actions, or refuse and risk access to their study population, potentially depriving them of the fruits of their work. And to the related point embodied in the disagreement between MSF-Belgium and Rennie, controversy persists as to whether cooperating with an authoritative body to study a practice in which they are engaged suggests support of that practice [55, 63]. Further exploration of these questions is essential as the role of research on humanitarian response expands.

Our results suggest that themes of cultural considerations, community engagement and mental health research incorporate ethical dilemmas related to cultural relativism. Accepting cultural norms such as gaining a husband’s consent for his wife’s participation in a research study, or excluding children from a research project on the grounds that including them is too high risk, equates to denying some of the fundamental principles of ethical research. Therefore, researching these populations may mean conceding to certain undesirable cultural norms and rejecting others that would require the researcher to compromise ethical standards. But where should the line be drawn? What guiding principles can future researchers employ? Bennouna et al.’s survey, which revealed most researchers claimed they would, if necessary, ignore local customs and include a child’s point of view in a study might help answer the question [67]. More of this type of research needs to be done in order to identify and resolve potential conflicts of local norms and traditional research ethics.

A surprising result of our study was that some researchers held the view that certain components of traditional, modern research ethics, such as formal consent, may be applied less rigidly in the humanitarian setting [21, 22, 44]. For example, arguments have been made that any consent is impossible in the case of experimental treatment for Ebola victims, and the failure to meet traditional standards should not preclude one from conducting this research [52]. On the other hand, there may be certain universal ethical principles of conducting research that should never be compromised. Exactly which principles these are, if any, have yet to be elucidated.

There are further unanswered questions with regard to the involvement of local institutions. Though our results point to a general agreement about the magnanimity of significant local involvement in research, including the development of local capacity for such work the inherent challenges have yet to be addressed [27, 33]. Humanitarian research is often conducted in places with little or no infrastructure and limited numbers of qualified researchers. Including local aid workers as researchers, solely for the inherent value of doing so, may prove costly and distract from other research mandates and aid delivery, particularly in disaster relief. As Tansey et al. put it, “while the global health research literature strongly endorses community engagement in all research, there have been few suggestions for overcoming challenges to carrying it out in the disaster setting” [33]. Future work must come to terms with this inevitable conflict of ideals.

Despite the unavoidable ethical challenges, the results of this systematic review suggest that not only is it possible to conduct research in this context, but there is an ethical obligation to do so [41, 48]. If the global community is compelled to provide assistance in the form of humanitarian action, than those in the humanitarian field must acknowledge the responsibility to develop rational, evidence-based approaches that are, at their core, ethically responsible [41]. This impulse is reflected in our results, which demonstrate an increasing number of publications on humanitarian research ethics since the inception of the Sphere project. The growing body of literature bodes well for researchers looking to ground their future work in a strong ethical foundation.

We would like to note, however, that the vast majority of articles included in this study were from high-income and Western countries. This highlights a finding in the research itself—that community participation and involvement of researchers from the countries and regions affected by crisis is limited. Addressing this inequity should be prioritized as the field of humanitarian research ethics progresses.

It should be noted that our study has limitations. We attempted to conduct a comprehensive review of the literature with a systematic review, augmented by known grey literature, but may have missed some potentially relevant literature that did not fit the search terms and was not identified via the grey literature review. This review is based primarily on published research literature and may exclude operational or programmatic reports with valuable insights. Also, though our initial search did include book chapters via the Scopus database, and dozens of chapters have been written on the subject, relatively few were screened into our final list of included literature. The reason for this is not immediately apparent. The authors did note a relative difficulty in the searching for and screening of book chapters when compared with other types of articles. This may have lead to a preferential selection of the latter type of literature, at the expense of the former.

The selection of papers was systematic and reproducible, and the analysis of those papers relied on standard qualitative methods. While the analysis may be considered less reproducible, we utilized a standardized interpretive methodology that would reliably highlight the critical findings and points within the papers as evidenced by the strong consensus between the authors (WB and RH) on almost every inclusion and exclusion decision. Though the limited literature base makes drawing firm conclusions difficult, the consistency of issues raised between and within the articles confirms the importance of the major themes elicited in this analysis.

## Conclusion

This study represents one of only very few attempts at a systematic review of research ethics in the humanitarian setting. We identified an increase in articles with robust ethical discussions particularly in the past few years. This promising trend could lead to further clarification and stronger ethical grounding of future research. Our data also highlight a number of unanswered questions related to fundamental conflicts that are unique to conducting research in the humanitarian setting. There is a clear need for further research and debate addressing these, and other important questions, such as: When is it appropriate to share data with local authorities? At what point should a researcher abandon a cultural relativistic point of view for an absolutist one? In a modern day humanitarian setting, what components of traditional ethics review may be anachronistic? How can researchers include local stakeholders as co-investigators when they may lack the training or infrastructure to do so? Mechanisms to translate these discussions into practical guidelines will need to be strengthened if the ideals of the Sphere Project are to be realized.

### Supplementary information

**Additional file 1: Supplementary Table 1.** PRISMA Checklist.

## Data Availability

The datasets generated and/or analyzed during the current study are available as tables in the manuscript.

## Appendix

**Search terms for systematic review of humanitarian research ethics**

1. *(Humanitarian OR “Global health”) AND (disaster OR emergency OR conflict) AND (ethic* OR bioethic* OR “human rights” OR rights) AND (research OR “program evaluation” OR “monitoring and evaluation” OR investigation) [MeSH terms].*
2. *(disaster) AND (ethic* OR bioethic* OR “human rights” OR rights) AND (research OR “program evaluation” OR “monitoring and evaluation” OR investigation) [MeSH terms]. Disaster medicine/ [MeSH]*

## Abbreviations

ERB: Ethical Review Board
LMIC: Low and Middle Income Countries
MSF: Médecins Sans Frontières
RCT: Randomized Clinical Trial
WHO: World Health Organization
